# Recent Advances in Electric-Double-Layer Transistors for Bio-Chemical Sensing Applications

**DOI:** 10.3390/s19153425

**Published:** 2019-08-05

**Authors:** Ning Liu, Ru Chen, Qing Wan

**Affiliations:** 1 Nanchang Institute of Technology, Nanchang 330099, China; 2 School of Electronic Science & Engineering, Nanjing University, Nanjing 210093, China

**Keywords:** ISFET, electric-double-layer, electrolyte-gated transistor, biosensor, chemosensor

## Abstract

As promising biochemical sensors, ion-sensitive field-effect transistors (ISFETs) are used widely in the growing field of biochemical sensing applications. Recently, a new type of field-effect transistor gated by ionic electrolytes has attracted intense attention due to the extremely strong electric-double-layer (EDL) gating effect. In such devices, the carrier density of the semiconductor channel can be effectively modulated by an ion-induced EDL capacitance at the semiconductor/electrolyte interface. With advantages of large specific capacitance, low operating voltage and sensitive interfacial properties, various EDL-based transistor (EDLT) devices have been developed for ultrasensitive portable sensing applications. In this article, we will review the recent progress of EDLT-based biochemical sensors. Starting with a brief introduction of the concepts of EDL capacitance and EDLT, we describe the material compositions and the working principle of EDLT devices. Moreover, the biochemical sensing performances of several important EDLTs are discussed in detail, including organic-based EDLTs, oxide-based EDLTs, nanomaterial-based EDLTs and neuromorphic EDLTs. Finally, the main challenges and development prospects of EDLT-based biochemical sensors are listed.

## 1. Introduction

Biochemical species detection techniques have important applications in the area of homeland security, medical and environmental monitoring, bioscience research and also food safety. In the past decades, many kinds of energy transduction principles have been employed for chemical and biological sensing based either on optical [1], electrochemical [2], electrical [3], gravimetric [4], acoustic [5], or magnetic methods [6]. Among these, electrochemical sensors provide an attractive means to analyze the content of target species due to the direct conversion of a biochemical event into an electronic signal. Sensors based on electrochemical transduction have been developed using a variety of techniques, such as chronoamperometry [7], chronopotentiometry [8], impedance spectroscopy [9], cyclic voltammetry [10], and various field-effect transistor (FET)-based methods [11], etc.

As a kind of potentiometric sensor, ion-sensitive field-effect transistors (ISFETs) are used widely in the growing field of biochemical sensing applications due to their advantages of label-free detection, easy miniaturization, integratability, robustness, etc. [12]. ISFETs were developed from metal oxide semiconductor field-effect transistors (MOSFETs) by replacing the metal gate electrode with a liquid solution and a reference electrode. In such devices, the charge carrier density in the semiconductor channel can be modulated over orders of magnitude by an electrostatic gating effect. Thus, the variation of surface potential on the sensing membrane arising from the adsorption of charged target species can be sensed by ISFETs. Due to the intrinsic property of signal amplification, ISFET devices are preferred for their low detection limits and high sensitivity, allowing them to outperform chemiresistors as well as amperometric sensors [13]. So far, a great deal of research effort has been directed toward the development of ISFETs with high sensitivity and specificity. In addition to sensing performance, flexible and stretchable ISFET devices with low power consumption are also in increasing demand in the emerging field of biosensing applications such as implantable chips, wearable electronics and point-of-care diagnostics [14,15,16].

The first generation of ISFET devices, proposed by Bergveld in 1970, were based on crystaline silicon (Si) MOSFET technology [17]. The main drawback of these devices is that their fabrication processes are relatively intricate and a high operating voltage (>10 V) is always required. Moreover, it is difficult to adapt Si-based ISFETs to curved surfaces or flexible structures due to the rigidity of Si substrate. Nowadays, large-area deposited thin-film transistor (TFT) technology, which is well compatible with flexible substrates, offers a lower cost alternative to produce ISFET sensors [18]. On the other hand, according to the sensing mechanism of ISFET, the change of surface potential induced by target species is reflected in a modulation of channel current. Thus, a strong electrostatic modulation at the dielectric/semiconductor interface is beneficial for the achievement of good sensing performance and low power consumption. Reducing the dielectric thickness and using a high-K gate dielectric are common methods that were used to obtain large capacitance of gate dielectric, and then to enhance the gate modulation. However, due to the direct tunneling of electrons, the gate leakage current will extremely increase with the reduction of the thickness of gate dielectric. Additionally, the adoption of high-K gate dielectrics will lead to a decrease in carrier mobility of channels owing to the electron trapping by Fröhlich polarons [19].

**Figure 1:**
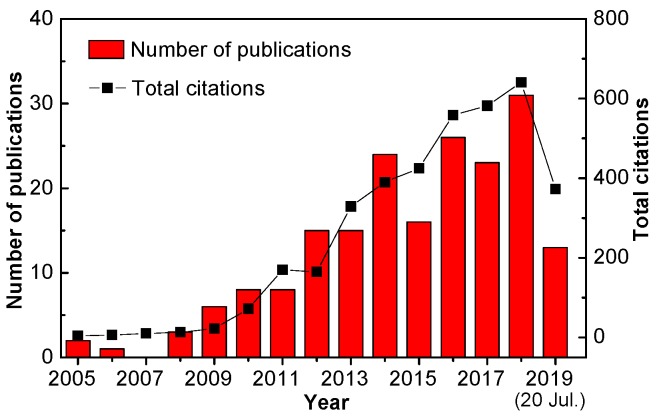
Number of publications and total citations on EDLT-based sensors sorted by year with key phrases “electrolyte-gated field-effect transistor sensor” or “electric-double-layer transistor sensor” searched in the Web of Science on 20 July 2019.

Recently, a new type of FET devices gated by ionic electrolytes has attracted intense attention due to their giant charge-carrier density accumulation induced by the extremely strong electric-double-layer (EDL) capacitive effect [20,21,22,23]. In such devices, electrolyte ions will migrate to the gate/electrolyte and semiconductor/electrolyte interfaces with the application of a gate voltage, functioning as two nanogap EDL capacitors with huge capacitance (>1 μF·cm^−2^) [24]. By the strong electrostatic EDL gating effect, a net charge accumulation/depletion in the semiconductor channel can be induced under a low gate voltage (<2 V). Characteristics of EDL-based transistors (EDLTs), such as large specific capacitance, low operating voltage, and sensitive interfacial properties, make these devices well suited to ultrasensitive portable sensing applications. To date, much effort has been devoted to the development of ultrasensitive biosensors with EDLT configurations, and remarkable sensing abilities have been demonstrated on various EDLT devices [25,26,27,28]. Figure 1 presents the development in the number and the total citations of EDLT-based sensor publications in the last fifteen years, showing an increasing interest in the topic.

In this review, we will focus on the recent progress and the performance of EDLT-based biochemical sensors. The concepts of EDL capacitance and EDLT are introduced first, and then the material compositions and the working principle of EDLT devices are described. In addition, the biochemical sensing performances of several important EDLTs, including organic-based EDLTs, oxide-based EDLTs, nanomaterial-based EDLTs and neuromorphic EDLTs, are discussed in detail. Finally, conclusions and outlook for the EDLT-based biochemical sensors are presented.

## 2. Physics of EDLTs: The Basics for Sensing

The key feature of an EDLT is the formation of EDLs at the gate electrode/electrolyte and electrolyte/semiconductor channel interfaces. The strong ion-induced EDL capacitive effect endows the EDLTs with high carrier density, low operating voltage and ultrasensitive interface property [20,29]. In this section, the EDL capacitance and the configuration of EDLTs are introduced at first, and then the materials in EDLTs, as well as the modulation and sensing mechanisms of EDLTs, are presented in detail.

### 2.1. EDL Capacitance and EDLTs

**Figure 2:**
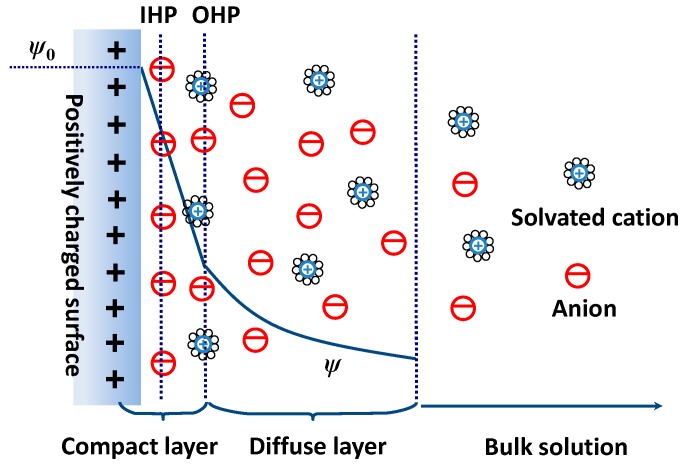
Stern model of the EDL generated on a positively charged electrode surface. Adapted from Ref. [30].

In 1853, the concept of EDL was first described by von Helmholtz to visualize the ionic environment in the vicinity of a charged surface. After several revisions, an EDL model proposed by Stern has been widely adopted in the investigation of EDL capacitances. Such model defines the EDL as two regions of ion distribution, i.e., a compact layer and a diffuse layer [30]. In the compact layer, the ions are strongly adsorbed by the electrode surface, consisting of an inner Helmholtz plane (IHP) and an outer Helmholtz plane (OHP). As shown in Figure 2, it is observed that IHP refers to the distance of closest approach of specifically adsorbed ions and OHP refers to that of the non-specifically adsorbed counterions. In the diffuse layer, which extends from the OHP to the bulk of the electrolyte, ions are distributed with a concentration gradient driven by the thermal motion.

According to the Stern model, the EDL capacitance can be treated as two capacitances in series, i.e., the compact double layer capacitance and the diffusion region capacitance. Typically, the capacitance of an EDL is governed by the capacitance of compact layer, in which the distance between the specifically adsorbed ions in electrolyte and the electrode surface is only a few Ångstroms [31]. Therefore, the EDL can act as a nanogap capacitor at the electrode/electrolyte interface. This EDL capacitor possesses high capacitance values (1–100 μF·cm^−2^), enabling strong electric field at the interface (up to 10^7^ V·cm^−1^) [32,33]. Meanwhile, a high carrier density up to 10^16^ cm^−2^ in the solid materials can be induced by the EDL capacitance [34]. In contrast, for usual oxide dielectrics, e.g., SiO_2_ and Al_2_O_3_, the capacitance is on the order of ~0.1 μF·cm^−2^ and the induced carrier density reaches a maximum of ~10^13^ cm^−2^ [35,36].

**Figure 3:**
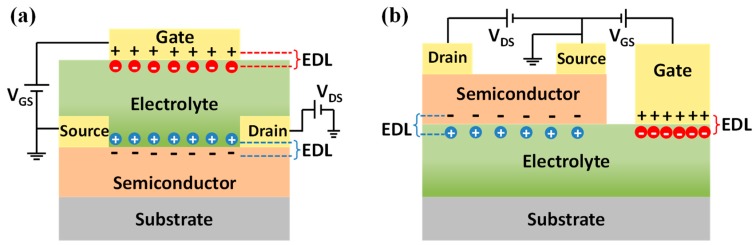
Schematic diagrams of (**a**) a top gate EDLT and (**b**) a side gate EDLT.

An EDL capacitor can be transformed into a transistor by replacing one electrode with a semiconductor layer which is contacted with source and drain electrodes. As it can be seen, the semiconductor channel is in ionic contact with a gate electrode via an electrolyte in the EDLTs (Figure 3a), which is different from the conventional transistor structures gated with oxide dielectrics. The carrier density of semiconductor channel can be effectively modulated by an ion-induced interfacial EDL capacitance. More importantly, the gate capacitance is decoupled with electrolyte thickness in EDLTs, enabling the use of thick, low-leakage electrolyte layers, as well as convenient side-gate geometries [37], as displayed in Figure 3b. According to the device configuration, an EDLT looks like an electrochemical transistor (ECT) [38,39,40]. However, in an ECT, the channel material is electrochemically active and permeable to the ions of electrolyte. The channel modulation is manipulated by the electrochemical doping/de-doping effect between electrolyte and semiconductor [41], which is essentially different from the operation of EDLTs dominated by electrostatic capacitive coupling effect.

### 2.2. Materials in EDLTs

The electrical properties of EDLTs are decided to a large extent by the characteristics of their active channels. Various semiconductor materials have been used as active channel layers in EDLTs, including organic semiconductors, oxide semiconductors, semiconducting nanomaterials, etc. Among these, organic-based EDLTs have significant advantages in low-cost fabrications and diversified surface groups, but their low carrier mobility (<1 cm^2^·V^−1^·s^−1^) and poor stability are unfavorable for ultrasensitive biospecies monitoring applications. Oxide-based EDLTs show relatively higher carrier mobility (~10 cm^2^·V^−1^·s^−1^) and better stability than those of organic devices, giving satisfactory sensitivity and sensing stability. Compared to the above two kinds of EDLTs, nanomaterial-based EDLTs exhibit highest sensitivity due to their small geometry and special electrical properties. However, the issues of low reproducibility and complicated fabrications should be further addressed. A detailed description of EDLT sensors based on different active channels is presented in Section 3.

In addition to active channels, the frequently-used electrolyte materials in EDLTs are introduced briefly. A wide spectrum of ionic materials have recently been reported as the gate electrolytes of EDLTs, including ionic solutions, polymer electrolytes, polyelectrolytes, ionic liquids and ionic gels, and inorganic solid electrolytes [29]. In the early stages, aqueous solutions composed of small ions, e.g., Na^+^, K^+^, Li^+^, Cl^−^, ClO_4_^−^, were used as the electrolyte dielectrics in EDLTs. For example, Heller and coworkers employed phosphate buffer saline (PBS) as the gate electrolyte in both the single walled carbon nanotube (SWNT) transistors and graphene transistors [42]. The responses of SWNT and graphene transistors to changes in the composition of the aqueous gate electrolyte were deliberately investigated. The results showed that the conductances of SWNTs and graphene were appreciably influenced by the ionic strength, the pH, and the type of ions present (K^+^, Li^+^, and tetraethylammonium ions). It was indicated that the manner of ionic gating was qualitatively different for graphene and SWNT devices. In SWNT-based EDLTs, the ionic gate affects the conductance primarily by changing the Schottky barriers at the interfaces between the SWNT and the source and drain contacts. However, in graphene-based EDLTs, the influences of ionic gating on the conductance are mainly due to the changes in number of charge carriers and their mobility. Noteworthy, ultrasensitive detections for certain biochemical reactions took place in aqueous solutions can be readily realized on the solution-gated EDLTs, rendering them quite suitable for biochemical sensing applications. Nevertheless, it is challenging to inhibit the electrochemical reactions between aqueous solutions and active channels, which are unfavorable for the robustness of EDLTs.

Besides aqueous electrolytes, polymer electrolytes prepared with different ions and polymer solvents have become one of the hottest electrolyte materials [43]. A perfect example is polyethylene oxide (PEO)/AClO_4_ (A = Li, K), in which ions A^+^ and ClO_4_^−^ dissolved in the polymer matrix can migrate freely under an electric field [44,45]. This type of polymer electrolyte is appropriate for various semiconductor materials, exhibiting a high EDL capacitance in the order of 10^−6^ F·cm^−2^. Moreover, electrostatic control of carrier density up to 10^15^ cm^−2^ in the organic semiconductor have been realized with the polymer electrolytes, which is much larger than those induced by conventional solid-state gate dielectrics [46].

Additionally, polyelectrolyte is another kind of polymer-based material exhibiting high ionic conductivity. Different from the polymer electrolyte in which both cations and anions are mobile under an electric field, only one ionic species is mobile in polyelectrolytes, while the charged polymer chains serve as immobile counter ions [47]. Typically, the movable ions in polyelectrolytes cannot penetrate into the bulk semiconductor, thereby the chemical doping will be suppressed effectively at the polyelectrolytes/semiconductor interface [41,48].

Most polymer-based electrolytes have slow ionic diffusion, making them relatively low switching speed for EDL gating [49]. Therefore, ionic liquids and ionic gels have been proposed as alternatives due to their faster ionic mobility and diffusivity [50,51,52]. Ionic liquids comprise nitrogen-containing organic cations and inorganic anions with high ionic concentration (~10^21^ cm^−3^). The electrostatic interactions between the component ions are relatively weak, endowing ionic liquids with high ionic conductivity [31]. Furthermore, ionic liquids display outstanding electrochemical stability in comparison to the other electrolytes. In spite of the excellent gating capabilities and fast responses, the “liquid” nature limits the utilization of ionic liquid in practical devices. As the derivatives of ionic liquids, ion gels are created by incorporating ionic liquid into engineered polymer networks, which have a solid-like mechanical integrity and preserve the inherent switching response of ionic liquids. With the advantages of high ionic conductivity, large specific capacitance and printability, ionic gels have been widely utilized as the gate electrolytes in EDLTs with excellent electrical and mechanical performances.

Apart from the above electrolyte materials, some inorganic films with microporous structure also exhibit high ionic conductivity, mainly due to the proton transport induced by the dissociation of adsorbed water in the high density micropores. A variety of inorganic electrolytes, such as nanogranular SiO_2_ and Al_2_O_3_, phosphosilicate glass, and zeolite, have been introduced as gate dielectrics in EDLTs [23,53,54,55]. In particular, the inorganic electrolytes are well matched with the oxide semiconductors due to the satisfactory interface quality. Excellent chemical stability and comparable large EDL capacitance (1–10 μF·cm^−2^) have been demonstrated on the oxide-based EDLTs gated by inorganic electrolytes.

### 2.3. Ion-Modulation in EDLTs

It is known that gating efficiency determines the level of accumulated carrier density in the FET. A high gate capacitance has been pursued to obtain high output currents and low operating power consumption. EDLT as a novel transistor are attractive for electronics technology due to the strong EDL gating effect and low-voltage operation. The operation mechanism of EDLTs is mainly based on the ion/electron (hole) electrostatic coupling effect, which can be explained as follows. When a voltage bias is applied to the gate electrode, the ions will move to the electrolyte/channel interface to form an EDL, thus image electrons (holes) can be induced in the semiconductor near the electrolyte/channel interface by an EDL capacitive effect. The induced charge density Q depends on the interfacial EDL capacitance (*C_DL_*) and the effective gate bias (*V_G_*) applied to the electrolytes in the following manner:

(1) $$Q=C_{DL}V_{G}$$

It is indicated that a high gate capacitance facilitates the induction of high-density accumulated carriers with a low gate voltage. Remarkably, even with a quite low gate bias of 1.0 V, the EDL capacitive coupling at the channel/electrolyte interface is still so strong, thus an electron (hole) accumulation layer can be induced to obtain a conducting channel.

**Figure 4:**
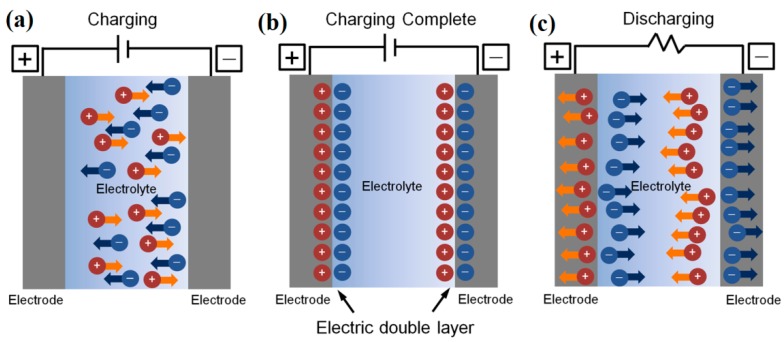
(**a**) The charge process in EDL formation under an external voltage. (**b**) The formation of EDL capacitors at the electrode/electrolyte interfaces. (**c**) The discharge process of EDL capacitors. Adapted from [29].

Apart from the strong electrostatic capacitive effect, EDLs exhibit unique charging kinetics attributed to the dynamic ion transports [56]. The formation of EDL under an external voltage can be divided into the charge process and the discharge process. Such processes can be explained as below. Initially, the mobile ions (anions and cations) are distributed randomly in the electrolytes. When an external voltage is applied to the electrodes, the anions in electrolyte will move towards the positive polarized electrode surface, while cations will be attracted to the negative polarized electrode surface, leading to a charge process (Figure 4a). Eventually, an EDL is formed at the electrode/electrolyte interface (Figure 4b). However, when the external voltage is removed, the discharge process starts until all ions drift back to the equilibrium position (Figure 4c). Usually, the mobility of ions is much lower than that of electrons, and then the ionic charge/discharge process for EDL formation will take a few milliseconds [57]. Therefore, the EDL capacitance is deeply dependent on the voltage frequency [58,59]. A stronger EDL capacitive effect will be observed in a relatively lower frequency range. The charging kinetics of EDLs can be affected by the electrode potential as well as the chemical composition and polarity of the ionic electrolytes [60]. Based on the dynamic ion modulation, unique transient characteristics can be observed on the EDLTs, which are mainly dominated by the ion migration within the electrolyte [61]. Additionally, the electrochemical doping of the semiconductor might occur when a high gate voltage is applied, which will further reduce the switching speed and the robustness of devices [62].

Despite the limitation of EDLTs in high frequency applications, the unique ion-induced EDL gating effect endows them with huge potential applications in many emerging fields wherein fast-switching operations are unnecessary, such as superconductivity [63], ferromagnetism [64], ferroelectricity [65], memory [66], biochemical sensing [67], and neuromorphic devices [68,69].

### 2.4. Sensing Principle of EDLTs

Based on the extraordinary high EDL capacitance, EDLTs have been widely used to develop biochemical sensors with low operation voltage and high sensitivity. During the sensing process, the target species adsorbed at the electrolyte interfaces can give rise to a change in the channel current of EDLTs, and as a result a sensing signal is observed. For instance, when a biochemical receptor is decorated on the interfaces, the reaction of the target species with the receptors can affect the interfacial potential and lead to a channel current response by an EDL capacitive coupling effect. To better understand the underlying physics, the operation of EDLT sensors can be dissected into two independent parts.

The first part is the sensing component, on which a change in the local electrostatic potential occurs due to the specific adsorption of target species. Both semiconductor channel and gate electrode can be used as the sensing element in the EDLT devices. The surface potential variation induced by analytes relies on the binding affinity of sensing membranes to target species. When an oxide-based sensing layer is used for pH sensing, for example, a hydroxylated surface on the sensing layer is formed due to the strong chemisorption of water [70]. Hydrogen ions in the solution can interacted with the hydroxyl surface to generate an interfacial potential. The surface potential *ψ*_0_ as a function of pH value has been deduced by [71] as:

(2) $$\frac{d\psi_{0}}{dpH}=2.303\alpha \frac{kT}{q}$$

where *q* is the elementary charge, *k* is the Boltzmann constant, *T* is the absolute temperature, and *α* is a dimensionless sensitivity parameter with a value between 0 and 1. It is indicated that the sensing membrane shows a maximum pH sensitivity of 59.5 mV·pH^−1^ at room temperature (300 K), which is known as the Nernst limit.

Many other interaction mechanisms between the analytes and recognition elements have also been developed for the signal generation [72], such as enzymatic reactions, ligand-receptor interactions, DNA hybridization, antigen-antibody affinity reactions, metabolic processes of living cells, etc. In either case, the key point is the surface charging of sensing membrane due to the adsorption of target charged species.

It should be noted that electrostatic interactions will be shielded by the ions in the electrolyte when a charge resides at a distance further than Debye’s screening length. This behavior defines the length-scale for electrical detections of charged analytes at the sensing interface [73]. In general, the Debye length is small in physiological solutions, hence, the probing of big size biomolecules would be seriously hindered [74]. Much effort has been made to address this critical issue, e.g., by decreasing the ionic concentration of test solutions [75], engineering the sensing surface [76] and minimizing the receptor sizes [77,78].

The second part of EDLT sensor is the EDLT-based transducer, on which the change in interfacial potential induced by target species can be converted into a change in channel conductivity by an EDL gating effect. When the gate electrode is used as the sensing element, a polarization of gate electrode can be induced by the charged species, which gives rise to a change in the effective gate potential. Such behaviors can be reflected by the variations in the threshold voltage (Vth) of EDLT device. According to the structure of such EDLT sensor, its Vth can be defined as [27]:

(3) $$V_{th}\left(EDLT\;sensor\right)=V_{th}\left(EDLT\right)+E_{ref}+\chi_{e}-\psi_{0}$$

As compared to the Vth of the original EDLT, three more factors contribute to the Vth of EDLT sensor, i.e., the constant potential of the reference electrode (E_ref_) applied to the solution of analyte, the interfacial potential surface dipole potential (χ_e_) on the interface, and the surface potential *ψ*_0_ on the gate electrode. Normally, *ψ*_0_ is a function of the amount of charged species adsorbed on the gate electrode, while the changes in χ_e_ are negligible in comparison to *ψ*_0_. Therefore, the changes of *ψ*_0_ with charged species can be detected by the variations in Vth, i.e., ΔVth ≈ −Δ*ψ*_0_.

However, when the semiconductor channel serves as the sensing element, the solution of analyte will be used as the electrolyte dielectric in the EDLT sensor. In this case, not only the interfacial potential, the EDL capacitance and even the carrier mobility at the semiconductor/solution interface could be influenced by the target charged species. This synergistic effect will lead to an obvious shift in the channel conductance. Therein, the sensitivity can be defined in terms of relative change in conductance [79], i.e., ΔG/G_0_ = (G − G_0_)/G_0_, where G_0_ is the channel conductance measured without target species. On the basis of the working principle of FET device, the sensitivity in the linear regime can be deduced as [80]:

(4) $$\frac{\Delta G}{G_{0}}=\frac{g_{m}\Delta \psi_{0}}{G_{0}}$$

while in the subthreshold regime it is then given by:

(5) $$\frac{\Delta G}{G_{0}}=exp\left(\frac{\gamma q\Delta \varphi_{0}}{kT}\right)-1$$

where g_m_ is the transconductance of the transistor device, and *γ* is the efficiency of gate coupling. In an EDLT sensor, the large EDL capacitance is beneficial for the channel modulation, leading to a large transconductance and a strong gating effect. Therefore, a high sensitivity can be easily achieved on the EDLT sensors.

In addition, the limit of detection (LOD) is another important sensing parameter, which is determined by the signal-to-noise ratio (SNR) of transducers. High SNR is desirable for recognizing extremely small signals. The SNR of an FET sensor depends on the transconductance and the intrinsic noise of the transistor [81,82]. A large transconductance facilitates a high SNR and then a low LOD of EDLT sensor. Moreover, the SNR can be improved by optimizing device geometry to obtain low-noise [83,84], and even by operating the transistor in the appropriate transport regime with lowest equivalent input noise [85].

## 3. EDLT Based Biochemical Sensors

Up to now, a wide range of EDLT devices have been investigated for biochemical sensing applications. Depending on the ion-induced gating effect of EDLT devices, new techniques have been developed to study biochemical interactions and overcome the physical limitations of ISFET technology. Here, the research progress on different sorts of EDLT sensors is reviewed. In Section 3.1 and Section 3.2, the state of art developments in EDLT sensors based on thin-film organic and oxide semiconductors are described, respectively. In Section 3.3, the sensing abilities of nanostructured EDLT sensors based on semiconducting nanomaterials, such as carbon nanotubes and graphene, are discussed. Finally, in Section 3.4, we introduce a novel sensing approach by using neuromorphic EDLT devices.

### 3.1. Organic-Based EDLT Sensors

More recently, organic semiconductors have received growing interests in flexible electronics and bioelectronics, thanks to their key features of synthetic freedom, solution processability, mechanical flexibility, biocompatibility, etc. [26]. Thus far, electrolyte-gated EDLTs based on various types of organic semiconductors, such as pentacene, poly(3-hexylthiophene) (P3HT), α-sexithiophene (α6T), have been widely studied for biochemical sensing.

**Figure 5:**
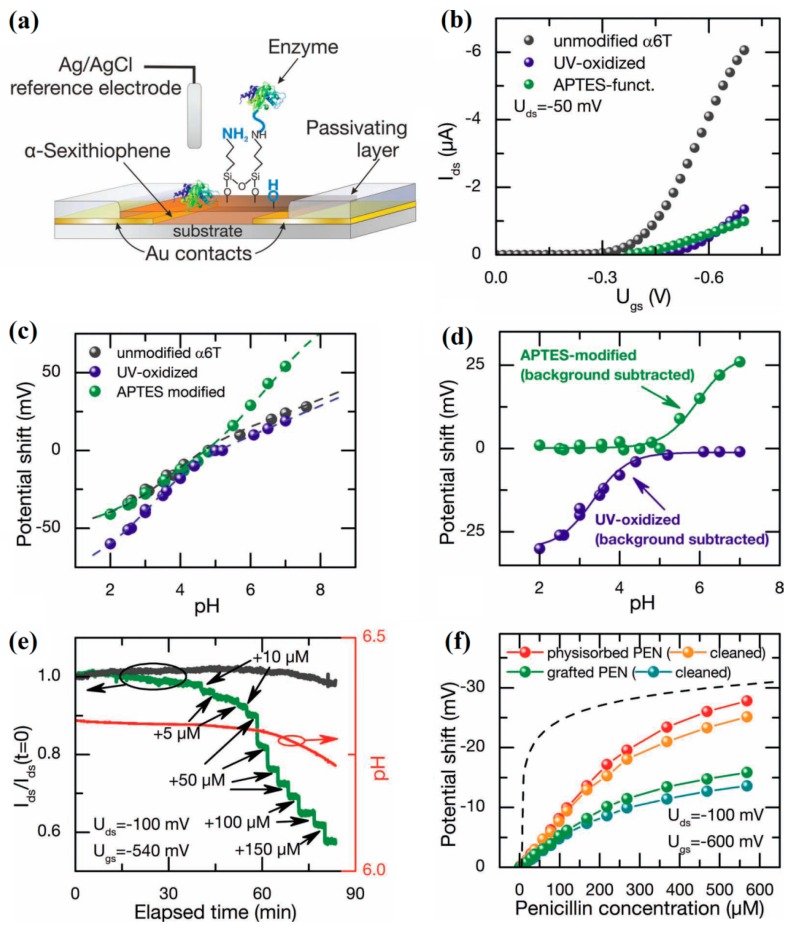
(**a**) Schematic of the solution-gated α6T-based EDLT sensor. (**b**) Transfer curves of unmodified, UV-oxidized and APTES-modified EDLTs, measured at pH 5. (**c**) Shifts in Vth of unmodified, UV-oxidized and APTES-modified EDLTs as a function of solution pH values. (**d**) Calibration of Vth shifts for APTES-modified device and oxidized device by subtracting the response of untreated α6T-based EDLT. (**e**) The responses of current with lapsed time to the addition of penicillin for the α6T-based EDLT functionalized with APTES/PEN (green line), and the untreated device (grey line). (**f**) Vth shifts against penicillin concentration for as-prepared devices with physisorbed enzymes, chemisorbed enzymes and after three washing steps. The dashed line indicates the simulated response of Vth shifts to pH variations in an unbuffered electrolyte. Adapted from [86].

In general, the organic semiconductor layer is synthesized with small molecules or polymer materials. The surface of organic materials can be easily tailored to covalently attach the recognition elements, thereby to identify different target species in aqueous solutions, for example some specific ions, glucose, DNA, proteins, and bacteria. Consequently, solution-gated organic EDLTs are particularly suitable for the detection of biochemical species in aqueous media. In this case, the conductivity of organic semiconductor channel, which is in direct contact with solutions, could be dramatically affected by the charged species at the organic channel/solution interface through an EDL capacitive effect. The solution acts both as the electrolyte dielectric and the media responsible for carrying the analytes. The sensing capabilities of such organic EDLTs rely on the specificity of the functionalized organic channels, on which the specific receptors can interact with the target species in the solutions. Buth et al. reported a solution-gated α6T-based EDLT in 2012 for pH sensing and penicillin detection [86], as shown in Figure 5a. For untreated α6T transistors, a sensitivity of 10–14 mV·pH^−1^ was obtained in the pH range from 2 to 8 (Figure 5c). Subsequently, the organic semiconductor surface was modified with hydroxyl by oxidation under UV or with amine groups by grafting (3-aminopropyl)triethoxysilane (APTES). Upon oxidation (300 s), an enhanced sensitivity of 26 mV·pH^−1^ was observed in the region below pH 5, while no changes were visible for higher pH values. In the case of APTES modification, however, the sensitivity increased to 21 mV·pH^−1^ in the neutral region but unchanged for more acidic solutions (Figure 5d). Furthermore, the penicillinase (PEN) enzyme was immobilized on the α6T channel to monitor the concentrations of penicillin (Figure 5e). It was found that only the PEN-modified device gave an obvious response to the addition of penicillin. Such sensor exhibited a sensitivity of 80 μV·μM^−1^ and a LOD of 5 μM for penicillin detection. Noteworthy, the APTES-treated devices did not show substantial advantages in both the sensing signal and the stability (Figure 5f).

Besides channel functionalization, the sensing receptors can also be immobilized on the gate electrode in the solution-gated EDLT sensors. Casalini et al. [87] described a potentiometric sensor based on a solution-gated P3HT-based EDLT for detection of dopamine (DA). In such organic EDLT, the selective and covalent binding between 4-formylphenyl boronic acid (BA) moiety and DA takes place on the gate electrode surface. A surface dipole potential can be induced by the covalent adsorption of DA molecules, resulting in a shift of gate electrode work function and a change of EDL capacitance. Consequently, a DA-dependent transfer characteristic was observed on the organic EDLT device with a lowest detection limit down to pM scale, which is three orders of magnitude lower than that of amperometric sensors based on the same sensing principle.

For the sensing of biological macromolecules, the effect of Debye screening for surface charges should be considered as a key point. Normally, the Debye length exhibits an inverse relationship with the ion strength of aqueous environment. Based on this principle, a water-gated organic field-effect transistor was proposed by Kergoat et al. for DNA detection [75]. They used a kind of organic materials called poly [3-(5-carboxypentyl)thiophene-2,5-diyl] (P3PT-COOH) as the semiconductor channel, onto which DNA probes were covalently grafted via NHS/EDC chemistry. Deionized water instead of more concentrated electrolytes was used in order to weaken the Debye screening effect. In this case, the DNA hybridization induced a decrease in the current of the organic EDLT sensors, whereas no significant change was observed when using PBS solution. However, such techniques are not suitable for in-site detections in actual biotic environment with high ionic concentration. 

**Figure 6:**
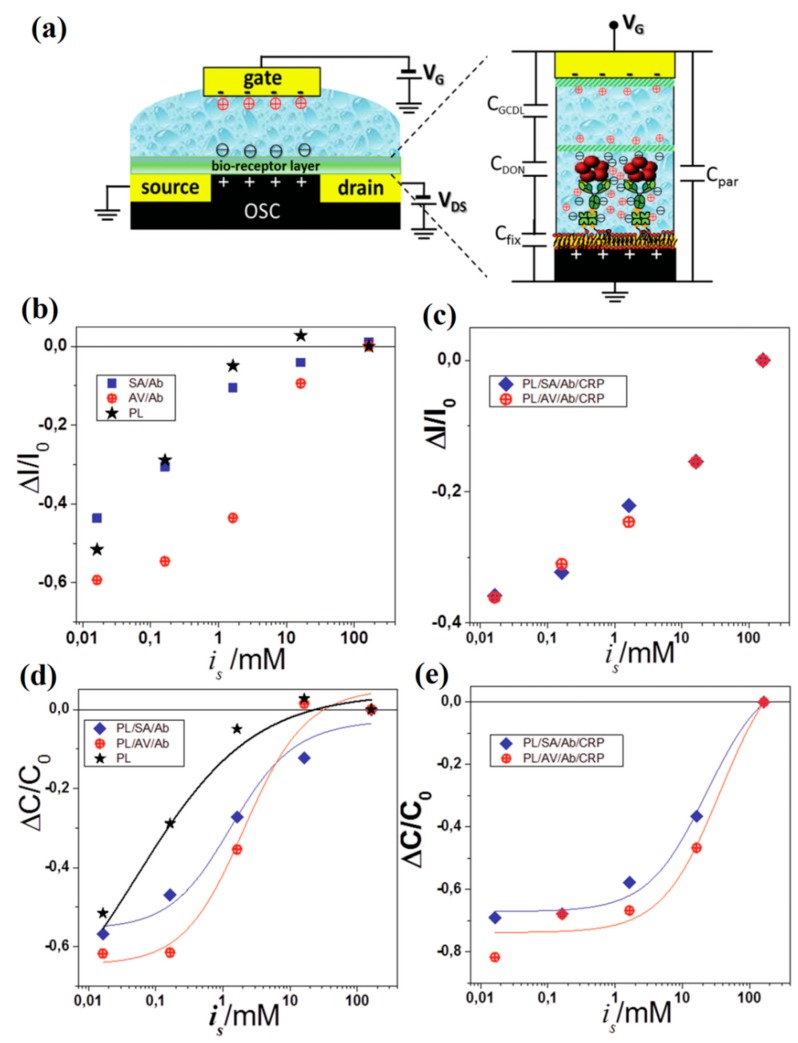
(**a**) Schematic of the solution-gated organic EDLT with the charge arrangements in a PL/SA/Ab/CRP multilayer shown on the right. The equivalent circuit of the capacitances involved is also shown. (**b**) Effect of the ionic strength on the relative current variations for the PL/SA(AV)/Ab and (**c**) PL/SA(AV)/Ab/CRP multilayers. (**d**) Effect of the ionic strength on the relative capacitance changes for the PL/SA(AV)/Ab and (**e**) PL/SA(AV)/Ab/CRP multilayers. Adapted from [88].

Palazzo et al. designed a solution-gated P3HT-based EDLT sensor by covalently anchoring a phospholipid (PL) bilayer to the organic channel [88]. Biotin/avidin (AV), biotin/streptavidin (SA) and C-reactive protein (CRP)/antibody (Ab) interactions were monitored on such sensor device, as shown in Figure 6a. The binding events taking place at a distance beyond the Debye length away from the organic channel were successfully detected in the presence of high salt concentrations (Figure 6b,c). The results are due to the formation of Donnan’s equilibria within the protein layers, leading to changes in gating capacitance at the electrolyte/organic channel interface (Figure 6d,e). This capacitive tuning effect is expected to enable the sensing responses of EDLTs immune to Debye screening.

Despite the good sensing performance and simple configuration of solution-gated organic EDLT sensors, a serious issue is that electrochemical doping and field-effect modulation often coexist in such devices [89,90], resulting in large hysteresis and poor stability during the sensing detection. To address this problem, Bao’s group fabricated a highly stable organic EDLT using a polyisoindigo-based polymer with siloxane-containing solubilizing chains33 (PII2T-Si) as the semiconductor channel [91]. Such organic EDLT can be used to monitor salinity changes and even be functionalized for the selective detection of heavy-metal ions in a seawater environment.

Keeping the organic channel away from the aqueous solutions is another alternative route for reliable sensing detection, which can be implemented on the organic EDLTs gated by solid-electrolytes. In a solid-electrolyte gated organic EDLT sensor, the organic channel does not need to be modified with selective receptor molecules, and it is not put in contact with the target-containing sample fluid. Instead, the biochemical interactions take place on the functionalized surface of gate electrode or solid electrolyte, which can also be transduced into a change of transistor characteristics by an EDL gating effect. Based on the strong EDL coupling effect, associating with the all solid state structure, novel transistor architectures, such as dual-gate structure, floating gate structure, etc., can be conveniently achieved on the solid-electrolyte gated EDLTs, facilitating the improvement of biochemical sensing capabilities.

**Figure 7:**
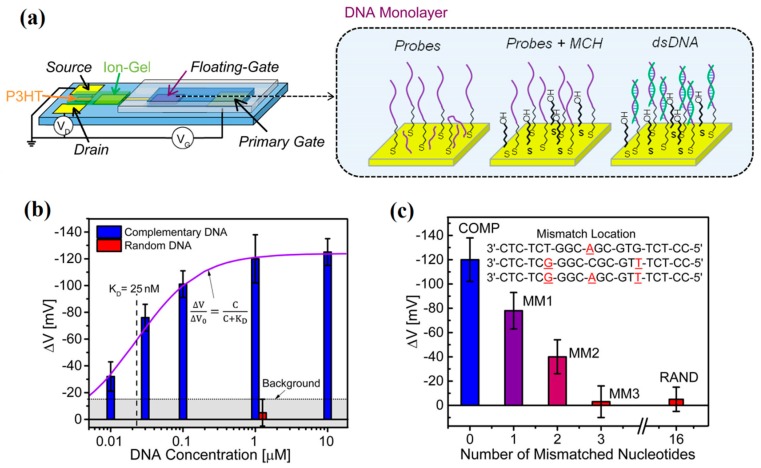
(**a**) Schematic structure of the floating-gate organic EDLT, in which the floating gate is modified with ssDNA probes and 6-mercaptohexanol (MCH), and then used to capture complementary DNA. (**b**) The sensitivity in terms of transfer curves shift (ΔV) as a function of the concentration of complementary DNA. The response is well fit by a Langmuir isotherm. The background level is defined as the sensor response to random DNA. (**c**) The sensor responses to complementary DNA, three kinds of mismatched DNA, and random DNA sequence, respectively. Adapted from [92].

Frisbie’s group explored a P3HT based EDLT gated by an ion-gel to sense the surface hybridization of DNA [92]. A floating-gate electrode functionalized with single-stranded DNA (ssDNA) was designed in such organic EDLT, as schematized in Figure 7a. The floating-gate potential is influenced by both the adsorption of DNA molecules and the capacitive coupling with a primary gate. When DNA is hybridized at the floating gate, the primary gate voltage will be partly offset, resulting in a change of channel current. A pronounced sensitivity in terms of Vth shift was obtained due to the extremely large transconductance of such EDLT (Figure 7b). Collective effects of surface dipoles and interfacial capacitances, arising from the chemisorption of target species on the floating gate, contributed to the sensor response of the floating-gate EDLT. The dependence of device architecture on the performance of such EDLT sensors was further investigated. The results demonstrated that floating-gate EDLT operation deeply depended on the relative areas of the semiconductor/electrolyte, floating gate/electrolyte, and control gate/electrolyte interfaces, which provided a valuable reference to the geometry optimization of EDLT devices for sensitivity enhancement [93].

Overall, a great many of organic EDLTs have been reported for biochemical sensors in detection of pH, ions, biomolecules and bioreactions, etc. Herein, many other more recent applications of organic EDLTs biosensors are summarized in Table 1 with particular emphasis on the materials category, device functionalization and sensing performances.

### 3.2. Oxide-Based EDLT Sensors

Oxide semiconductors, especially amorphous ones, are a promising category of channel materials for EDLTs attributed to the merits of high optical transparency, relatively high electron mobility, low temperature deposition, low-cost large area and good stability. A variety of semiconducting oxides have been used as channel layers in EDLTs, including zinc oxide (ZnO) [103], indium oxide (In_2_O_3_) [104], indium zinc oxide (IZO) [105], indium tin oxide (ITO) [106], and indium gallium zinc oxide (IGZO) [107]. Compared to organic EDLTs, EDLTs based oxide semiconductor permit stable detection under harsh environments due to their electrical and thermal robustness. Moreover, metal oxides are superior sensing materials for biochemical detections. Hence, much effort has been made on oxides-based EDLT sensors for achieving ultrasensitive, low-power and robust sensing applications.

In typical oxide-based EDLT sensing systems, oxide semiconductors can act simultaneously as the sensing element and the active channel of FET transducer. Chae et al. [108] presented an amorphous IGZO-based solution-gated EDLT as a biological sensing platform. Such IGZO-EDLT devices showed perfect uniformity of threshold voltage with a small chip-to-chip deviation when exposed to deionized water environments. After modification of IGZO channel with APTES, the device showed a higher sensitivity of 68.5 mV·pH^−1^ in terms of Vth shifts in comparison to that (32.7 mV·pH^−1^) of the bare IGZO-EDLT. Furthermore, monoclonal antibody molecules were chemically immobilized on the IGZO surface for the capture of target proteins. Subsequently, the functionalized IGZO-EDLTs were used to monitor the presence of alpha-synuclein (αS) proteins in the solution. A depletion of electrons and a corresponding decrease in conductance of the EDLTs were observed due to the adsorption of negatively charged αS proteins at the IGZO/solution interface. A linear sensitive response of 9.35 mV·dec^−1^ was obtained to αS proteins in concentration ranges from 10 fg·mL^−1^ to 1 ng·mL^−1^, verifying the applicability of IGZO-EDLTs for biochemical sensing detections.

**Figure 8:**
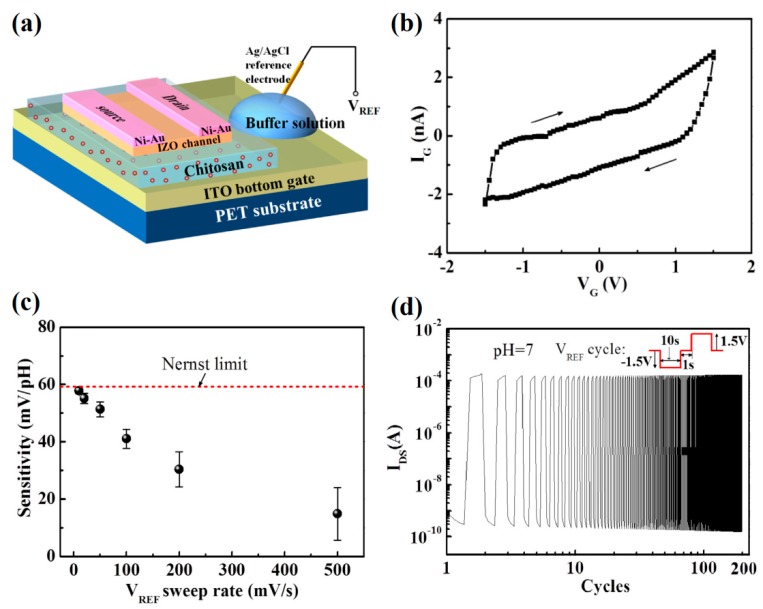
(**a**) Sketch map of the chitosan-gated IZO-EDLT sensor. (**b**) Leakage curve of the chitosan electrolyte film. (**c**) The sensitivity of the chitosan-gate EDLT as a function of the gate sweep rate. The red dash line indicates the Nernst limit. (**d**) Stability test driven by square-wave gate voltages of −1.5 V to 1.5 V applied to the pH 7 solution at V_DS_ = 0.1 V. Adapted from [109].

Due to the relatively smooth surface, oxide semiconductor layers exhibit remarkable compatibility with solid electrolytes, enabling them suitable for configuring all-solid state EDLTs, which have better robustness and more flexible geometry. Recently, our group proposed a flexible all-solid state IZO-based EDLT for pH sensing, as displayed in Figure 8a [109]. An acid doped chitosan-based biopolymer layer was employed as the gate dielectric. A large EDL capacitance of 5.4 μF·cm^−2^ at 1 Hz was observed at the chitosan/IZO interface. The bottom ITO gate electrode served as the sensing element in contact with pH solutions. Such an IZO-EDLT sensor showed a high sensitivity of ~57.8 mV·pH^−1^ close to Nernst limit when operated at a slow gate sweep rate of 10 mV·s^−1^ (Figure 8c). More importantly, a low gate leakage current below 3 nA and robust ionic modulation were demonstrated in the chitosan-gated EDLTs (Figure 8b,d). The same type oxide-EDLTs gated by nanogranular SiO_2_ electrolyte were also successfully used to detect dopamine molecules [110], avian influenza virus H5N1 [111], and immune molecules [112], by immobilizing special receptors on the electrode gate or oxide channel, respectively.

**Figure 9:**
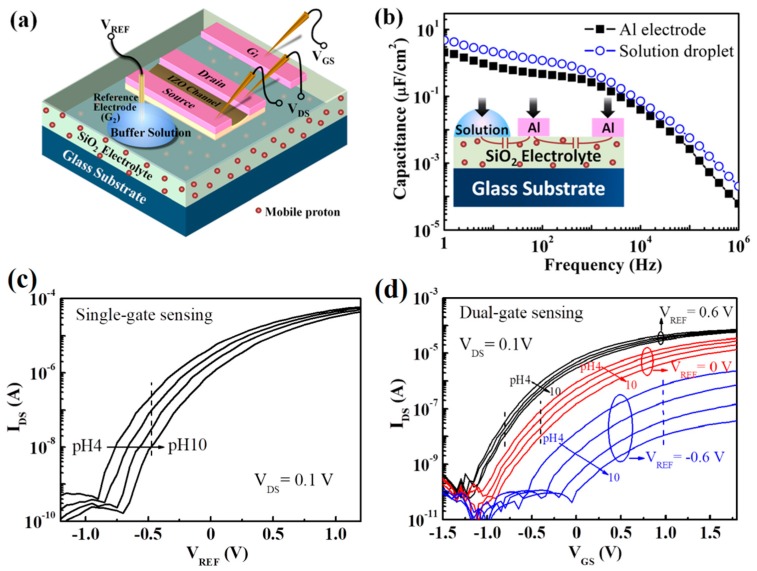
(**a**) The schematic diagram of the laterally coupled dual-gate IZO-based EDLT sensor. (**b**) The specific capacitance vs frequency curves of the SiO_2_ electrolyte films for metal gate and solution gate (pH = 6.0), respectively. Inset: the lateral gate test structure. (**c**) pH-dependent transfer characteristics of the laterally coupled IZO-EDLT in single-gate sensing mode. (**d**) pH-dependent transfer characteristics of the laterally coupled IZO-EDLT in dual-gate sensing mode at different V_REF_ of −0.6, 0, and 0.6 V, respectively. Adapted from [113].

Another advantage of the all-solid state oxide-based EDLTs is that it is convenient to realize some novel transistor architectures on such devices, such as co-plane dual-gate structures. An enhanced sensitivity can be achieved on these novel dual-gate EDLTs. Wan’s group developed a laterally coupled dual-gate IZO-based EDLT for pH sensing application [113]. Figure 9a shows the schematic diagram of such a dual-gate EDLT sensor. It is observed that the channel and source/drain, gate electrodes of the device are all located on the nanogranular SiO_2_ electrolyte film. The carrier density of the IZO channel can be effectively modulated by two lateral gates, i.e., a metal primary gate and a solution sensing gate, respectively. A higher EDL capacitances of ~4.8 μF·cm^−2^ was obtained for sensing gate compared to that (~2.0 μF·cm^−2^) for primary gate with a lateral gate structure (Figure 9b). In single gate sensing mode, as shown in Figure 9c, the device exhibited a sensitivity of ~48 mV·pH^−1^. The sensitivity can be further enhanced by an amplified capacitive coupling ratio between the sensing gate and the primary gate. Eventually, an extremely high sensitivity of 168 mV·pH^−1^ beyond Nernst limit was achieved at a liquid gate bias (V_REF_) of −0.6 V in dual-gate sensing mode on such IZO-EDLTs (Figure 9d). Laterally coupled dual-gate EDLT provides a facile strategy for high-performance biochemical sensing devices.

### 3.3. Nanomaterial-Based EDLT Sensors

With the rapid development of nanomaterials and nanofabrication techniques [15], great interest in nanomaterial-based EDLT sensors has been aroused due to their features of ultra-small size, high surface-to-volume ratio and particular physicochemical properties, which are significantly different from their bulk counterparts. A multitude of nanostructure EDLTs have been tailor-made for ultrasensitive biochemical detection applications. Examples of such nanomaterials include carbon nanotubes (CNTs), graphene, molybdenum disulfide (MoS_2_) nanosheet, various inorganic nanowires, etc. Massive classes of detection scenarios, such as glucose, DNA, cell, bacterial, and protein sensing, have been broadly investigated based on the nanomaterial-based EDLTs [28]. Table 2 summarizes the recent reports on the sensing performances of diverse nanomaterial-based EDLT sensors. As a representative, here the development of EDLT sensors based on CNTs and graphene is introduced emphatically.

Since the discovery of CNTs in 1991 by Sumio Iijima [125], these one-dimensional carbon nanomaterials have emerged as building blocks for novel nanoelectronic devices [126]. Based on their unique physical, chemical and electrical properties, CNTs have been widely investigated as potential candidates for development of next generation miniaturized biosensors [127]. 

As early as 2002, Rosenblatt et al. fabricated a SWNT-based transistor gated by a NaCl solution [128]. It was found that the gate electrolyte had little impact on the mobility of the SWNT, and the electrolyte/SWNT capacitance was near the quantum capacitance. Ultrahigh carrier mobilities (~1000 cm^2^ V^−1^s^−1^), low contact resistances, and high transconductances were demonstrated on such devices. The excellent device characteristics of the SWNT-based EDLTs make them ideal for biosensing applications. Since then, CNTs have been functionalized with specific receptors to be capable of recognizing various biospecies by a solution-gated EDLT strategy [129,130,131]. In most cases, a charged target species near the CNT acts as an effective gate, and then tunes the conductance of CNT by an electrostatic coupling effect.

**Figure 10:**
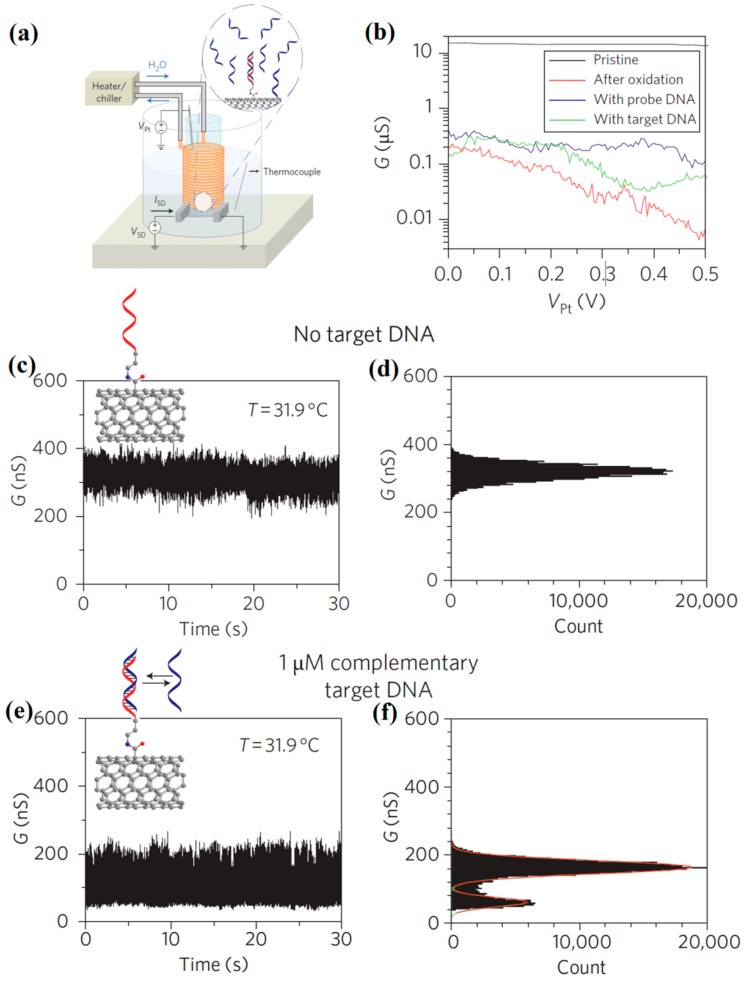
(**a**) Schematic measurement setup of the solution-gated CNT transistor sensing platform. (**b**) Conductance of CNT vs V_Pt_ at different stages. (**c**) Conductance recordings of DNA probe modified CNT transistor immersed in PBS without complementary DNA target. (**d**) Conductance-based histograms of time intervals extracted from the data in (**c**). (**e**) Conductance recordings of DNA probe modified CNT transistor immersed in PBS with 1μM complementary DNA target. (**f**) Conductance-based histograms of time intervals extracted from the data in (**d**). The two levels are fitted by Gaussian distributions. Adapted from [132].

Thanks to their nanoscale dimensions, that are comparable to those of typical biomolecules, CNTs should be able to electrically perceive single biomolecules. Besides, the large transconductances of CNT-based EDLTs facilitate the identification of small signals from single molecules. In 2011, Sorgenfrei et al. realized the detection of DNA hybridization at a single-molecule level using solution-gated CNT transistors [132]. In such CNT sensor, a probe ssDNA sequence was covalently attached to a carboxylic acid modified point defect in a CNT. Then, this device was employed to investigate the kinetics and thermodynamics of DNA hybridization at certain temperatures. The schematic measurement layout is shown in Figure 10a. The conductance of CNT against the potential on a platinum gate electrode (V_Pt_) was measured at different stages, i.e., before oxidation, after oxidation, after overnight coupling with probe DNA and after exposure to target DNA (Figure 10b). The results showed that the original CNT was metallic, but exhibited an obvious gate response after oxidation. For real-time measurements in the absence of target DNA (Figure 10c,d), it was observed that the conductance of the solution-gated CNT transistors fluctuated around 300 nS. The conductance fluctuations were absolutely dominated by flicker (1/f) noise. When a small quantity of complementary target DNA was added into the gate solution, however, a decreased conductance with large amplitude two-level fluctuations appeared, as displayed in Figure 10e,f. Such behaviors are mainly attributed to the increased scattering and charge transfer at the defect position arising from target DNA binding. By fitting the fluctuations to a two-level model, it was concluded that the modulation in conductance of CNT was dominated by only a single DNA attachment. The kinetics of DNA hybridization was further investigated on such device, demonstrating non-Arrhenius behavior of the system, which agreed with the measurements using fluorescence correlation spectroscopy. This technique is powerful to probe single-molecule dynamics with fast time resolution.

Lately, two-dimensional (2D) nanomaterials have been exploited for biomedical sensing with fascinating properties. Graphene is a typical 2D material comprised of a single layer of carbon atoms with a honeycomb structure [133]. Taking the advantages of extremely high carrier mobility, low intrinsic electrical noise, and large specific surface area, great progress has been made on exploiting graphene devices for various classes of biosensors [134,135,136,137]. Among these, solution-gated graphene transistor has shown to be the most promising one. Such devices are expected to exhibit ultrahigh sensitivities based on the merits as follows. Firstly, the 2D nanostructure enables every atom of graphene fully exposed to the solution environment, leading to a comprehensive interaction between the graphene and the target species in solutions, which renders them high-performance sensors. Secondly, due to the large transconductances and high carrier mobilities, a great response of channel current in the solution-gated graphene transistor can be induced by a small amount of charged species on the graphene surface, which is favorable for the signal amplification in sensing detections. Thirdly, owing to its exceptional high mobilities and ambipolar field-effect characteristics, solution-gated graphene transistor shows unique radio-frequency properties [138]. By using a measuring strategy at high frequencies, an improved sensitivity is expected on account of the suppression of charge screening effect.

In 2008, Ang et al. firstly fabricated a solution-gated graphene transistor using epitaxial graphene grown on silicon carbide substrates [139]. Such a device was measured in a standard electrolyte of 10 mM PBS solution whose pH values can be further adjusted by adding 0.5 M hydrochloric acid or potassium hydroxide solution. Ambipolar transfer behaviors were observed in the solution-gated graphene transistors. The hole and electron mobility reached a maximum of 3600 and 2100 cm^2^·V·s^−1^, respectively. An obvious positive shift in transfer curves was obtained with the increase of pH, resulting in an over-Nernstian response of 99 mV·pH^−1^. By further investigation on the electrochemical properties of the EDL on graphene, it was confirmed that the pH-sensitive behavior of the solution-gated graphene transistors arose from the capacitive charging of the ideally polarizable graphene/electrolyte interface.

Due to the great biocompatibility of graphene, solution-gated graphene transistors have been developed in wide applications of biosensing detections. After certain modifications, solution-gated graphene transistors could achieve similar or higher sensitivities to biospecies compared with other sensing techniques [140,141]. For example, Chen et al. proposed a monolayer graphene-based transistor for DNA hybridization detections [142]. In such device, the conductance of the probe DNA modified graphene can be modulated by a gate bias applied to a PBS solution containing target DNA molecules. A negative shift in charge neutrality point (V_CNP_) was observed with an increase in concentration of target DNAs. Compared to the few-layer graphene devices, the V_CNP_ shift of single-layer graphene FETs was enhanced by 140%. Moreover, a low buffer concentration is favorable for the sensing responses of solution-gated graphene to DNA attachments. In addition, the cleanness of graphene surface had a significant impact on the interaction between DNA and graphene. Finally, an optimum sensitivity as low as 1 pM for DNA detection was achieved on the solution-gated monolayer graphene-based transistor.

**Figure 11:**
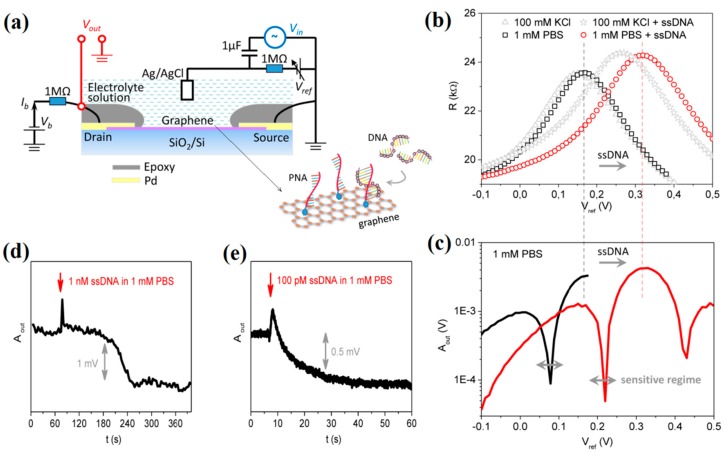
(**a**) Schematic diagram of an electrolyte-gated graphene transistor operated in a frequency-doubling mode. In sensing process, the hybridization of negatively charged ssDNA with peptide nucleic acid (PNA) anchored on graphene surface occurs. (**b**) Transfer characteristics of electrolyte-gated graphene transistors before and after ssDNA adsorption in 1 mM PBS or in 100 mM KCl solutions, respectively. (**c**) Responses of A_out_-V_ref_ characteristics for electrolyte-gated graphene transistors to the ssDNA adsorption in 1 mM PBS. Recordings of A_out_ in real time upon the addition of (**d**) 1 nM and (**e**) 100 pM ssDNA into 1 mM PBS, respectively. Adapted from [143].

Recently, there is also growing interest in applying radiofrequency mode in solution-gated graphene transistors for biochemical sensing applications. A low detection limit and ultrafast responses are expected to be realized on the graphene radio-frequency device attributed to the wide bandwidth and significantly reduced 1/f noise at high frequencies. Recently, a frequency-doubling technique was implemented on the electrolyte-gated graphene transistor for biochemical detections [143]. Figure 11a shows the configuration of electrolyte-gated graphene transistor operated in a frequency-doubling mode. In such device, an input sinusoidal voltage applied to the electrolyte gate can be rectified to a sinusoidal wave at the drain electrode with a frequency gain. Due to the high carrier mobility and the strong electrolyte gate coupling, the graphene frequency doubler exhibited a high output energy efficiency more than 95% and an unprecedented unity gain. High stability against drift and superior noise performance were also demonstrated on such devices. As a proof of concept, such electrolyte-gated graphene transistors were used for detecting nonspecific ssDNA molecules adsorption by frequency-doubling operation. Figure 11b displays the transfer curves recorded before and after adding ssDNA into 1 mM PBS buffer solution. It was observed that the resistance increases from 23.6 kΩ to 24.3 kΩ at the V_CNP_, in addition to a positive V_CNP_ shift of 150 mV. The results implied that the carrier scattering slightly increased because of the adsorption of ssDNA on the graphene surface. Furthermore, the liquid gate voltage (V_ref_) dependent second harmonic output (A_out_) was measured, as shown in Figure 11c. In the linear operational regime, the amplitude of A_out_ was relatively small but varies steeply with the change of V_ref_. Remarkably, a relative change of A_out_ up to 8 times was observed upon ssDNA adsorption in the sensitive regime, which was 2 orders of magnitude higher than that of the traditional measurements (~8%, as shown in Figure 11b). The real-time responses of the graphene frequency doubler to the addition of ssDNA were recorded in Figure 11d,e. Depending on the drift-free performance and the reduced low-frequency 1/f noise by sampling at doubled frequencies, an ultralow detection limit down to ~4 pM was achieved for ssDNA molecules monitoring.

In short, nanostructured EDLTs have been successfully used in various types of biochemical sensing platforms. Owing to the high specific surface areas and the strong interactions with charged species, nanostructured EDLT sensors exhibit better sensing performance compared to their bulk counterparts, enabling ultrasensitive biochemical detections, such as single-molecule recognition and single-cell monitoring. However, several critical issues, including device-to-device variations and complicated fabrications, still need to be addressed for further promotion of nanostructure EDLT sensors in real applications.

### 3.4. Neuromorphic EDLT Sensors

As we enter a new age of intelligence, biochemical sensors are being developed towards miniaturization, monolithic integration and ultralow power. New special functions, such as high throughput analysis, multicomponent identification and temporal-spatial resolution, are desirable to be implemented on the individual sensor chip with low power consumption.

The human brain is a sophisticated biological nervous system, which can carry out complex computations with low power dissipations base on their highly parallel and event-driven running mode [144]. There is an increasing interest in pattern recognition with artificial neuromorphic devices due to the merits of parallel information processing, low-power consumption, self-learning and robustness [145,146,147]. In neural network, two neighboring neurons are connected by a synapse, which undertakes signal transmitting and basic information processing by ionic fluxes regulations [148]. Thus, the realization of biological synaptic functions on a physical device is essential to build artificial neuronal networks. Nowadays, EDLT devices have been explored for neuromorphic emulations based on their ion-induced EDL modulation and unique dynamic electrical characteristics [149,150,151]. Synaptic functions and neural network information processing can be implemented on the EDLTs with very low power consumption.

For EDLT-based artificial synapses, their synaptic behaviors are quite sensitive to the ion transport characteristics in electrolyte, as well as the external stimulation applied on the gate. Wan et al. proposed an IGZO-based EDLT gated by different types of liquid solutions to mimic synaptic behaviors. Typical neural information processing functions based on short-term synaptic plasticity were realized on these EDLTs, including paired-pulse facilitation, high-pass filtering and orientation tuning. More importantly, the short-term synaptic plasticity can be effectively modulated by adjusting the alcohol or salting strength in gate solutions. This behavior is attributed to the influence of salt ions on the delivery of hydronium and hydroxide ions in forming of EDL. These results pave the way for neuromorphic EDLTs in the applications of ultralow power biochemical detections.

Inspired by the spike stimulation and dendritic integration in biological neurons, Liu et al. proposed a flexible oxide-based neuromorphic EDLT with multi in-plane gates for pH sensor applications [152]. Due to the proton migration within nanogranular SiO_2_ electrolyte, such device presented distinctive time dependent transient electrical characteristics. The single-spike responses of the neuromorphic EDLT sensors to different pH solutions were investigated. 

**Figure 12:**
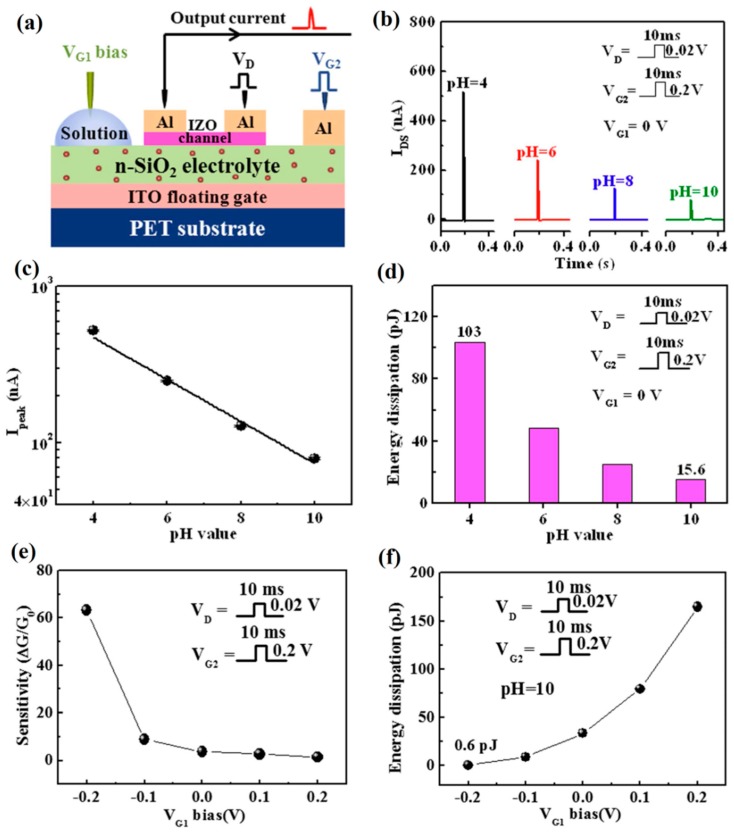
(**a**) Scheme map of spike sensing measurements on an oxide-based EDLT device. (**b**) The spike response to different solutions with pH value increasing from 4 to 10. (**c**) The logarithm of current peak as a function of solution pH value. (**d**) pH-dependent energy dissipation in single-spike sensing mode. (**e**) The variations in sensitivity and (**f**) energy dissipation (pH = 10) against different gate bias applied on pH solutions. Adapted from [152].

Figure 12a describes the single-spike pH sensing measurement on a neuromorphic EDLT. In the measurement, a constant gate bias was applied on the solution gate, while the ion equilibrium was broken by a small voltage spike applied on the metal gate, leading to a pH-dependent transient current response (Figure 12b,c). A rapid recovery of current response was observed when the spike was finished. The fast spike response facilitated low power pH sensing detections, as displayed in Figure 12d, a low power consumption ranging from 15.6 pJ/spike to 103 pJ/spike was obtained with a decreasing pH value from 10 to 4. Due to the capacitive inter-coupling effect, an enhanced sensitivity and a lower power consumption were achieved when a more negative voltage bias was applied on solution gate (Figure 12e,f). Compared with the conventional quasi-static sensing mode, the single-spike sensing mode exhibited higher pH sensitivity, faster sensing response and lower power consumption. These results confirmed the feasibility of EDLT sensors in neural spike mode. However, the dependence of target species on the synaptic plasticity of EDLT devices was not studied, which is crucial for ultralow-power neuromorphic sensing applications.

**Figure 13:**
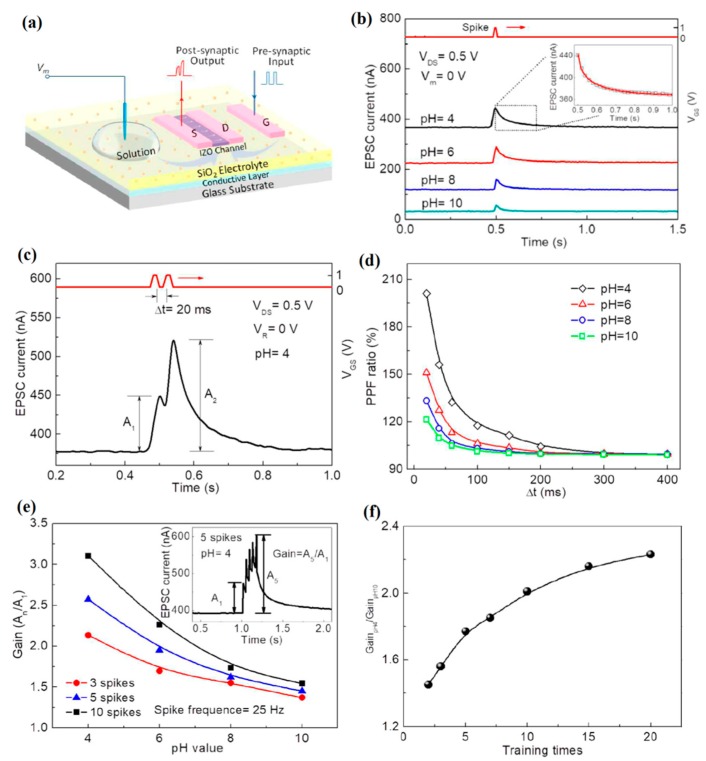
(**a**) Sketch map of IZO-based synaptic EDLTs. (**b**) Channel current responses to voltage spike (1 V, 10 ms) under different pH solutions. Inset: the enlarged view of current decay curve at pH = 4, which is well fitted with a stretched exponential function. (**c**) Response of EPSC to two successive presynaptic spikes (1 V, 10 ms) with a time interval of 20 ms at pH = 4. (**d**) PPF ratios as a function of time intervals under different pH solutions. (**e**) EPSCs amplitude gains (A_n_/A_1_) against pH values measured with different spike numbers. Inset: the response of EPSC to five successive V_GS_ spikes (1 V, 10 ms) at pH = 4. (**f**) The relative amplitude gains (Gain_pH4_/Gain_pH10_) between pH = 4 and pH = 10 as a function of training times. Adapted from [153].

More recently, the same research group further investigated the pH dependent synaptic responses of IZO-based synaptic EDLTs gated by nanogranular SiO_2_ electrolyte films [153]. The schematic diagram of an IZO-based synaptic EDLT is displayed in Figure 13a. 

In the measurement of the synaptic emulation, voltage spikes applied on the Al gate electrode acted as the pre-synaptic inputs, while a fixed gate bias was applied on the pH buffer solutions. The channel current response was considered as the post-synaptic output, which was recorded with a constant reading drain voltage. It was observed that the response of channel current to a voltage spike started with a rapid increase, and then declined slowly to the base line. This behavior is similar to the excitatory post-synaptic current (EPSC) in nervous system. Due to the pH dependent surface charging of SiO_2_ electrolyte, regular changes in EPSC amplitude, retention time and base current with different pH value were obtained on the IZO-based synaptic EDLTs (Figure 13b). In addition, the typical synaptic plasticity, such as paired-pulse facilitation and short-term potentiation, can be regulated by different pH solutions (Figure 13d,e). Base on the pH dependent synaptic responses, such device exhibited an increasing pH sensitivity in terms of relative gain of EPSC amplitude between pH = 4 and pH = 10 by multi-spike trains (Figure 13f). These studies provide an intriguing new-concept for neuromorphic biosensors based on EDLT synaptic devices.

## 4. Conclusions and Outlook

In this review, we have presented recent advances in EDLTs applied in biochemical sensors. The operation of EDLTs is based on the ion-induced capacitive coupling effect at the electrolyte/ semiconductor interfaces. Due to the strong EDL capacitance, the electrical properties of EDLTs depend deeply on the charged species at the interface, making EDLTs more sensitive than common ISFET devices for biochemical sensing applications. A great deal of effort has been devoted to achieving high sensitivity, selective detection, robustness, and low power consumption for EDLT-based sensors. The research progresses on different sorts of EDLT sensors were described in details.

All in all, the investigation of ELDT-based sensors in the past decade has achieved great success, and will continue to be exciting and highly rewarding. Nevertheless, there are still significant challenges. Firstly, the selection of materials in EDLT sensors should be well-directed based on a profound understanding of their chemophysical properties at the solid/solid or solid/liquid interface. Secondly, an important issue concerning the integration of biochemical receptors onto the EDLT devices needs to be addressed, without degradation of their activity. The modification of sensing surface should be developed specifically for different EDLT sensors. Thirdly, most ELDT devices reported for sensing measurements are operated in strictly controlled conditions with little consideration of the various interference factors widely existing in real environments. Thus, the sensing performances of the ELDT-based sensors should be further characterized and optimized in practical testing circumstances. Finally, compared to the mature integrated technology for ISFETs, the integration of EDLT-based sensor with digital processing unit and power source should be further investigated, which is of great importance for the fabrication of sensor microchips that can be used in actual sensing applications.

In the future, there will be much innovating and developing space for EDLT sensors. First of all, the possibilities of processing EDLT sensors by printing technologies could make their fabrication particularly cost effective for mass production [40]. In addition, owing to the low-voltage/low-power operation and the strong compatibility with flexible substrates, EDLT-based sensors exhibit huge potential in the field of Internet of Things applications, such as wearable medical devices, implantable sensor chips, real-time environment analyzing, etc. [31]. More importantly, based on the inherent properties of dynamic ion modulation, novel architectures and innovative test methods could be exploited on EDLT-based sensors, making it feasible for multiplex sensing and high-resolution temporal-spatial detection. It is expectable that some EDLT-based sensors will become mature enough to enjoy scientific and commercial success in the near future.

## Figures and Tables

**Table 1:** Main characteristics of some types of organic EDLT sensors.

Channel	Electrolyte	Target Species	Receptor/Immobilization Site	Carrier Mobility (cm^2^·V^−1^·s^−1^)	Sensitivity/LOD	Selective Detection	Refs.
P3HT	10^−2^ M NaCl solution	Na^+^	Polymeric ion selective membrane/electrolyte	0.02	62 mV·dec^−1^	Less sensitive to K^+^	[94]
P3HT	PBS solution	Procalcitonin (PCT)	BSA + anti-PCT antibody/channel	0.001–0.01	LOD is 2.2 pM	No response to milk powder	[95]
P3HT	High pure water	C-reactive protein (CRP)	BSA + anti-CRP/gate electrode	nr	LOD as low as 13 ± 4 proteins	No response with sole BSA	[96]
DPP-DTT	PBS solution	Cu^2+^	Gly-Gly-His peptide/ gate electrode	nr	1 mA·dec^−1^; LOD is 1 pM	Less sensitive to Fe^2+^, Mn^2+^	[97]
DPP-DTT	Water	2,4-dichlorophenoxyacetic acid (2,4-D)	2,4-D-C1-alkyne + antibody/ gate electrode	~1	LOD is 2.5 fM	No response to 2,4,5- trichlorophenoxyacetic acid	[98]
Pentacene	PBS solution	TNFα	Peptide aptamers/gate electrode	nr	LOD is 1 pM	No response to interleukin-6	[99]
Pentacene	PBS solution	Plum Pox Virus (PPV)	Protein G + anti-PPV antibody/gate electrode	nr	LOD reaches sub ng·mL^−1^	No response with anti-TNFα antibody	[100]
pBTTT	PBS solution	Bisphenol A (BPA)	Alkyl-BPA + antibody/channel	nr	LOD is 2 pg·mL^−1^	Less sensitive to dibutyl phthalate	[101]
P3CPT	10^−1^ M NaCl + MES solution	Histamine	H_2_ histamine receptor/channel	nr	LOD is 1.6 nM	Less sensitive to putrescine, tyramine and histidine	[102]

nr: not reported; BSA: bovine serum albumin; TNFα: tumor necrosis factor alpha; MES: 2-(N-morpholino)ethanesulfonic acid; DPP-DTT: poly(N-alkyldiketopyrrolopyrrole dithienylthieno[3,2-b]thiophene); pBTTT: Poly(2,5-bis(3-tetradecylthiophen-2-yl)thieno[3,2-b]thiophene); P3CPT: poly{3-(5-carboxypentyl)thiophene-2,5-diyl}.

**Table 2:** Sensing performances of diverse nanostructured EDLT sensors.

Nano Channel	Gate Electrolyte	Target Species	Receptor	Carrier Mobility (cm^2^·V^−1^·s^−1^)	Sensitivity/LOD	Selective Detection	Refs.
Silicon nanowire	PBS solution	Prostate specific antigen (PSA)	APTES + PSA antibody	nr	LOD is 1.5 fM	nr	[80]
Silicon nanowire	PBS solution	Dopamine released from Living Cells	DNA oligonucleotide of 57-mer	nr	LOD is 1 fM	No response without cells seeding	[114]
ZnO nanowire	pH buffer solutions	H^+^	APTES	1.85	~90 mV·pH^−1^	nr	[115]
ZnO nanorods	PBS solution	glucose	Glucose oxidase	nr	LOD is 3.8 μM	nr	[116]
SWNTs	PBS solution	Dopamine	nr	21.1	LOD is 10^−18^ M	nr	[117]
SWNTs	PBS solution	Epsilon toxin (ETX)	PBSE + ETX antibody	nr	LOD is 2 nM	nr	[118]
CNTs	PBS solution	Acetylcholine	Acetylcholinesterase	nr	5.7 µA·dec^−1^	No response to serine and glycine	[119]
CVD-grown graphene sheet	PBS solution	Complementary DNA	Probe DNA	nr	LOD is 0.01 nM	Less sensitive to mismatched DNA	[120]
Reduced graphene oxide	PBS solution	Acetylcholine	Acetylcholinesterase	0.5	ΔI/I_min_ = 1.06 dec^−1^ in the range of 0.1–10 mM	nr	[121]
Reduced graphene oxide	PBS solution	Ca^2+^, Hg^2+^	Calmodulin; metallothionein type II protein	nr	LOD is 1 nM	No response to lake water	[122]
Single-layer graphene	PBS solution	Complementary DNA	Probe DNA	nr	24 mV·dec^−1^; LOD is 25 aM	Less sensitive to SNP	[123]
MoS_2_ nanosheet	PBS solution	Streptavidin	Biotin	nr	I/I_0_ = 196 at 100 fM	nr	[124]

nr: not reported; APTES: (3-aminopropyl)triethoxysilane; PBSE: 1-pyrenebutanoic acid succinimidyl ester; SNP: single nucleotide polymorphism.
